# Nano-confined crystallization of organic ultrathin nanostructure arrays with programmable geometries

**DOI:** 10.1038/s41467-019-11883-6

**Published:** 2019-09-02

**Authors:** Hanfei Gao, Yuchen Qiu, Jiangang Feng, Shuang Li, Huijie Wang, Yuyan Zhao, Xiao Wei, Xiangyu Jiang, Yewang Su, Yuchen Wu, Lei Jiang

**Affiliations:** 1 Key Laboratory of Bio-inspired Materials and Interfacial Science, Technical Institute of Physics and Chemistry, Chinese Academy of Sciences, 100190 Beijing, People’s Republic of China; 2 University of Chinese Academy of Science (UCAS), 100049 Beijing, People’s Republic of China; 3 College of Chemistry, Jilin University, 130012 Changchun, People’s Republic of China; 4 State Key Laboratory of Nonlinear Mechanics, Institute of Mechanics, Chinese Academy of Sciences, 100190 Beijing, People’s Republic of China; 5 School of Engineering Science, University of Chinese Academy of Sciences, 100049 Beijing, People’s Republic of China; 6 Key Laboratory of Bio-inspired Smart Interfacial Science and Technology of Ministry of Education, School of Chemistry, Innovation Institute of Frontier Science and Technology, Beihang University, 100191 Beijing, People’s Republic of China; 7 Beijing Key Laboratory of Engineered Construction and Mechanobiology, Institute of Mechanics, Chinese Academy of Sciences, 100190 Beijing, People’s Republic of China

**Keywords:** Structural properties, Synthesis and processing, Two-dimensional materials

## Abstract

Fabricating ultrathin organic semiconductor nanostructures attracts wide attention towards integrated electronic and optoelectronic applications. However, the fabrication of ultrathin organic nanostructures with precise alignment, tunable morphology and high crystallinity for device integration remains challenging. Herein, an assembly technique for fabricating ultrathin organic single-crystal arrays with different sizes and shapes is achieved by confining the crystallization process in a sub-hundred nanometer space. The confined crystallization is realized by controlling the deformation of the elastic topographical templates with tunable applied pressures, which produces organic nanostructures with ordered crystallographic orientation and controllable thickness from less than 10 nm to *ca*. 1 μm. The generality is verified for patterning various typical solution-processable materials with long-range order and pure orientation, including organic small molecules, polymers, metal-halide perovskites and nanoparticles. It is anticipated that this technique with controlling the crystallization kinetics by the governable confined space could facilitate the electronic integration of organic semiconductors.

## Abstract (web summary)

Fabrication of ultrathin organic semiconductor nanostructures with precise alignment, tuneable morphology and high crystallinity remains challenging. Here the authors use an assembly technique with dewetting process controllability for patterning organic single-crystal arrays in a sub-hundred nanometer space.

## Introduction

Ultrathin organic materials are considerable opportunities, as well as challenges for applications in the electronic and optoelectronic devices, owing to their strong quantum confinement, long-range-ordered molecular packing, solution processability and mechanical flexibility^1–6^. Various ultrathin organic nanostructures have been fabricated with the development of different preparation methods, such as interface synthesis^7–9^, epitaxial growth^10–12^, and blade-casting technique^13^. The confined growth of crystal in two dimensions (2D) is accessible by two approaches, which are the employment of molecules with planar configuration, such as some 2D metal-organic frameworks (MOFs) and covalent organic frameworks (COFs)^3,9,14,15^, and the introduction of strong epitaxial interactions. For instance, an interfacial polycondensation reaction has been employed for fabricating a long-range ordered monolayer COF film^16^. The van der Waals interaction epitaxy has also been demonstrated to assemble few-molecular-layer single crystals of 2,7-dioctyl[1]benzothieno[3,2-b][1]benzothiophene (C_8_-BTBT)^2^. However, large-area ordered nanostructures are needed for the integration of organic electronic devices, thus it still remains challenging to fabricate high-performance ultrathin organic patterns with precise alignment, high-quality crystallography and tunable morphology.

Recently, for the integration of organic architectures, various solution patterning techniques have been established such as inkjet printing^17–19^, nanoimprinting lithography^20–22^, capillary-bridge lithography^23,24^, dip-pen lithography^25–27^, and solution-shearing technique^28–30^. These techniques realize the confined crystallization of organic crystals through patterning micro-droplets, leading to the accurate control of crystal position and morphology. However, the regulation of the crystallization process in all three dimensions, especially in the height direction, is still restricted. For conventional patterning techniques, the height of crystals was influenced by the quantity of molecules, but cannot be continuously tuned. Therefore, it has a limitation on continuously tuning the morphology of organic single crystals in a large area, especially for the patterning of few-molecule-layer nanocrystals, for the high-quality integration of organic electronics.

In this work, an efficient patterning technique is developed for fabricating of ultrathin organic single-crystal arrays with high crystallinity, ordered crystallographic orientation and programmable morphology. The nucleation and growth of organic molecules are implemented in the confined space, thus leading to the fabrication of one-dimensional (1D) single-crystal arrays. With the tunable applied pressure, the thickness of the confined space is controlled by the spontaneous variation of liquid volume considering the increasing stress of the liquid layer, which is analyzed by the mechanical simulation. Therefore, the height tailoring of organic belts arrays is operated, yielding the large-area ultrathin 1D arrays with continuous tunability in height from less than 10 nm to approximately 1 μm. Organic ultrathin nanostructured arrays are assembled with different shapes induced by confined crystallization, including circle, circular ring, square, four-sided ring, pentagon, five-sided ring, hexagon, and six-sided ring. We have further demonstrated the general application of our method by employing organic small molecules, polymers, metal-halide perovskites and nanocrystals for patterning ultrathin nanostructures with long-range order and pure crystallographic orientation.

## Results

### Fabrication of 1D ultrathin organic arrays

To prepare the confined space for the fabrication of 1D ultrathin organic arrays, a photoresist micropillar template was manufactured through the photolithography technique. The SU-8 photoresist was employed considering its superior mechanical properties, self-smoothing, high thermal stability and corrosion resistance^31^. Micropillar templates were fabricated by the ultraviolet (UV) photolithography (schematically illustrated in Supplementary Fig. 1). Morphological characterization by scanning electron microscopy (SEM) confirms the uniform size (*ca*. 5 μm in width and *ca*. 12 μm height), accurate alignment, smooth surface of photoresist micropillars (Supplementary Fig. 2a, b). The fabrication condition is optimized by statistics of the formation ratio of micropillars with different lengths, widths and heights (Supplementary Fig. 2c, d). Note that developing process is crucial for the formation ratio of templates, during which the capillary force of developer solution can cause varying degrees of damage for micropillars with different height/width/length ratios^32^. The anti-swelling property of photoresist micropillars to solvents, such as toluene, is significant for the preparation of organic semiconductors. To validate the anti-swelling property, the micropillars were immersed in toluene for 24 h and less than 1% swelling ratio was observed (for pillars of over 2 μm in width) (Supplementary Fig. 3). Therefore, the photoresist micropillar template is demonstrated with designable architecture, swelling resistance compared with the common flexible template of polydimethylsiloxane (PDMS) replica and low cost compared with silicon pillar template.

**Fig. 1:**
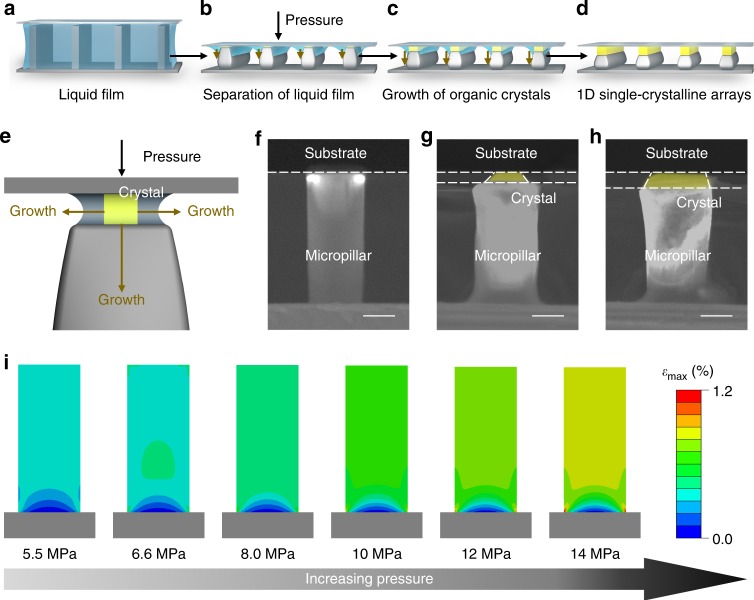
Controlled dewetting process for assembling 1D organic arrays. **a**–**d** Schematic illustration of 1D organic arrays assembly processing under pressure. **e** Zoom-in schematic illustration of 1D organic crystal nucleus growing in the confined space. **f**–**h** ESEM images with cross-sectional view of organic nanostructure growing on an individual micropillar, showing the assembly under pressure. **i** Simulated maximum principal strain of a typical photoresist pillar. Strain of the micropillar increasingly exhibits at the bottom, especially for associative part of the pillar and silicon substrate with pressure increasing from 5.5 to 14 MPa. Scale bars: **f**–**h** 3 μm

To pattern the 1D ultrathin organic arrays, a layer of 6,13(bis-triisopropylsilylethynyl)pentacene (TIPS-pentacene) solution was sandwiched between photoresist micropillars and a target flat substrate (Fig. 1a, Supplementary Fig. 4). By applying a static pressure between micropillars and the substrate, the distance (<100 nm) between tops of micropillars and the target substrate is reduced considering the elasticity of the photoresist (Fig. 1b). With the evaporation of solvents in the fabrication process, the thin liquid film contracted towards the gaps between micropillars’ tops and substrate for the formation of nano-confined capillary bridges, which is driven by the Laplace-pressure difference^33^. Further evaporation of solvents in nano-confined capillary bridges induced the pinning and receding of gas-liquid-solid three-phase contact line (TCL). When the organic solution become supersaturated, organic crystals nucleated and grew in the nano-confined gap (Fig. 1c). The gaps with a serval-nanometer height tend to restrict the vertical growth of crystals, while growth of crystal keeps free along other two dimensions, yielding ultrathin nanobelt arrays (Fig. 1d, e). The growth direction of organic crystals is determined by the direction of micropillars, which is controlled by unidirectional capillary flows in nano-confined capillary bridges. To study the crystal-growth behavior dominated by the elastic template, we observed the cross-section morphology of organic crystals and photoresist micropillars at different stage through environmental scanning electron microscope (ESEM). A relatively low pressure of 8.0 MPa was applied to monitor the response of micropillars in the crystallization. The growth of crystal was stopped at different stage by adding N,N-dimethyformamide (DMF) to remove the residual solution. As shown in Fig. 1f–h, the constriction of photoresist pillars occurs synchronizing with the crystal growth in the vertical direction. This phenomenon suggests that the strain of elastic micropillars introduced by the vertical growth of crystals can impede the crystallization, which illuminates us to control the growth of ultrathin nanobelts by applying a large pressure.

In our experiments, the applied pressure was optimized between 5.5–14 MPa for controlling growth of nanobelts (detailed measurement of pressure in this system was shown in Supplementary Fig. 5). To rationalize the influence of pressure on photoresist micropillar templates, we firstly simulated the mechanical performance of micropillars under different pressures. We employed finite element analysis (FEA) to calculate the distribution of strain, stress and displacement of a typical micropillar (details shown in the Methods Section; model illustrated in Supplementary Fig. 6a, b). As shown in Fig. 1i and Supplementary Fig. 6–8, micropillars maintain stable geometry with smooth and straight outline within 14 MPa, suggesting that the stability of capillary bridges at different applied pressures in our experiments. The FEA results also indicate the nearly linear deformation of micropillars along the height direction with the increase of pressure, which is also illustrated by ESEM images (Supplementary Fig. 9). We secondly conducted buckling simulations of the system in order to compute the critical pressure of structural instability. The first-order buckling mode exhibits the feasible morphology of a photoresist micropillar under the critical pressure of 39 MPa, which is much greater than the selected pressure range, thus demonstrating the reliability of our method (Supplementary Fig. 10a). In our experiment, the fracture of micropillars occurs under pressure exceeding 32 MPa (Supplementary Fig. 10b).

### Morphological and crystallographic characterization

**Fig. 2:**
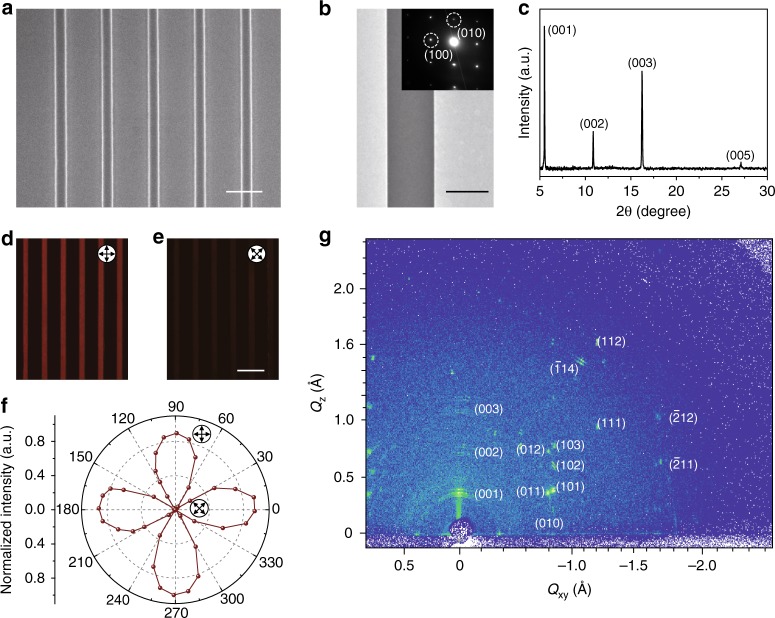
The morphological and crystallographic characterization of 1D organic arrays. **a** SEM image of 1D organic single-crystal arrays with precise alignment and regular morphology. **b** TEM image of an individual organic microbelt with straight boundary and high-quality crystallinity shown in SAED pattern of the inset. **c** Powder XRD pattern of 1D arrays, conforming to the oriented triclinic TIPS-pentacene with the space group of *P*1 and lattice constant of *a* = 7.6 Å, *b* = 7.8 Å, *c* = 16.8 Å, *α* = 89.2°, *β* = 78.4°, and *γ* = 83.6°. **d**–**e** Parallel-polarized and cross-polarized reflection images of 1D arrays. **f** Polarizing angle dependent reflection intensity of 1D arrays, demonstrating their high-quality crystallinity and uniform crystal orientation. **g** GIWAXS pattern of 1D arrays, indicating the crystal orientation of 1D arrays along with [010]. Scale bars: **a** 10 μm, **b** 1 μm, **e** 20 μm

For evaluating the quality of as-prepared TIPS-pentacene nanobelt arrays, we employed the SEM, atomic force microscopy (AFM). As shown in Fig. 2a, SEM image manifests a series of high-quality organic nanobelts with the distance of *ca*. 10 µm assembled in an array with negligible misalignment angle (<1°), homogeneous width of *ca*. 2.5 µm, while the smooth surface of nanobelts is demonstrated by the SEM and AFM images of an individual nanobelt with low root mean square roughness (*R*_q_) of 0.416 nm (for a 1.0 × 1.0 µm^2^ area) (Supplementary Fig. 11a, c). Transmission electron microscopy (TEM) image illustrates no detectable grain boundary (Fig. 2b). Selected area electron diffraction (SAED) pattern with the crystal faces of (010) and (100) and dihedral angle of 89.2° suggests the growing direction of the 1D single-crystal arrays along with crystal orientation of [010], which is consistent with the *π*–*π* stacking direction and benefits carrier transport^34^. X-ray diffraction (XRD) was employed for demonstrating the crystallography of 1D arrays. The XRD diagram (Fig. 2c) manifests a series of peaks of 5.50°, 10.86°, 16.22°, and 27.10°, which can be assigned to (001), (002), (003), and (005) diffraction (*a* = 7.6 Å, *b* = 7.8 Å, *c* = 16.8 Å, *α* = 89.2°, *β* = 78.4°, *γ* = 83.6°, space group, *P*1). This suggests the preferential [001] crystallographic orientation in out-of-plane direction^35^. Parallel-polarized and cross-polarized reflection images of 1D arrays gained by the polarization microscopy evidence the pure crystallographic orientation of 1D arrays (Fig. 2d–f).

To further assess the crystallography and analysis the molecular packing of 1D arrays, the grazing-incidence wide-angle X-ray scattering (GIWAXS) was harnessed (the Methods Section). The GIWAXS pattern illustrates the sharp and discrete Bragg diffraction spots, demonstrating the high-quality single crystallinity of 1D organic arrays (Fig. 2g). The distinct diffraction spots of (01L), (10L), (11L), and (−21L) indicate the highly controlled orientation of 1D aligned single-crystal arrays^28,29,36^. As a comparison of these high-crystallinity and oriented 1D arrays, the ultrathin organic films fabricated by spin-coating method present rough surface with a high *R*_q_ of 3.26 nm (for a 1.0 × 1.0 µm^2^ area) and a large quantity of grain boundary as shown in the SEM and AFM images (Supplementary Fig. 11b, d). XRD diagram of the thin film illustrates the Bragg diffraction peaks of 5.52°, 10.86°, 12.92°, and 16.22° corresponding with the faces of (001), (002), (011), and (003) with low signal-to-noise ratio, while its GIWAXS pattern shows the diffraction ring, implying the polycrystallinity and disordered orientation (Supplementary Fig. 12).

### Size control of 1D arrays

**Fig. 3:**
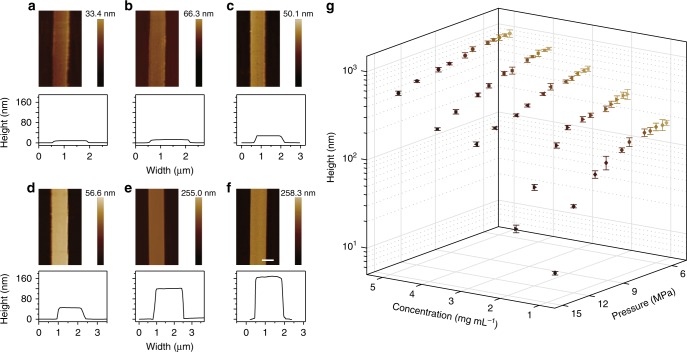
The size control of 1D organic arrays by tuning pressure and solution concentration. **a**–**f** AFM images of an individual organic single-crystal nanobelt with the width of *ca*. 1.5 μm but different heights varying from 8.3 nm to 168.1 nm. Their operating conditions are **a** and **b** 1 mg mL^−1^ 14 MPa, **c** and **d** 1 mg mL^−1^ 12 MPa, **e** 1 mg mL^−1^ 8.0 MPa, **f** 1 mg mL^−1^ 6.0 MPa, respectively. **g** Tunable thickness of nanobelts at different solution concentrations varying from 1 to 5 mg mL^−1^ under different pressures varying from 5.5 to 14 MPa. Scale bars: **f** 1 μm

For tailoring the size of organic belt arrays, the solution concentration and applied pressure are both imperative due to their capability for regulating the nucleation and growth of organic molecules. To conclude the influence of concentration for regulating the size of organic nanobelts, we proposed explanation of dewetting and crystallizing mechanism, and then provided the statistics with concentration changing. The nucleation of organic crystal in the dewetting process is mainly dominated by the solution concentration. For solution with high concentration, the nucleation and growth of organic crystals occur in the confined space with a larger size in accordance to the fluid dynamics simulation reported in our previous work^33^, then yielding the lengthened growth of crystal for obtaining the enlarged single crystalline belts (Supplementary Fig. 13a). Therefore, nanobelts in width of 1.6–3.9 µm and height of 175.3–871.9 nm were fabricated by precursor solution with various concentrations of 1–5 mg mL^−1^ (Supplementary Fig. 13b–e).To gain the ultrathin nanostructures, the pressure is applied using different-concentration solutions. Through tuning the pressure and concentration, the nanobelt arrays with heights ranging from 8.3 to 932.0 nm were fabricated (Fig. 3a–f). According to the lattice parameter of TIPS-pentacene crystals, the height of 8.3 nm corresponds to 5 layers of moleclues, validating the successful fabrication of ultrathin nanobelts. AFM images explain that ultrathin nanobelts with the height of less than 80 nm exhibit the imperfect surface morphology with rough edges and surfaces, whereas the nanobelts with the height more than 80 nm exhibit high quality with sharp edges and smooth surfaces. Ultrathin nanobelts growth at low concentration under high pressure could be limited by the lack of solute in the confined space, which does not benefit the nucleation and growth of crystalline grains, thus leading to the generation of grain boundary and surface defects. To understand fabrication of organic ultrathin nanobelts induced by solution concentration and pressure, we carried out the statistics of height of nanobelts (average height of 5 nanobelts for each point) fabricated by concentration ranging from 1 to 5 mg mL^−1^ and pressure ranging from 5.5 to 14 MPa (Fig. 3g).

**Fig. 4:**
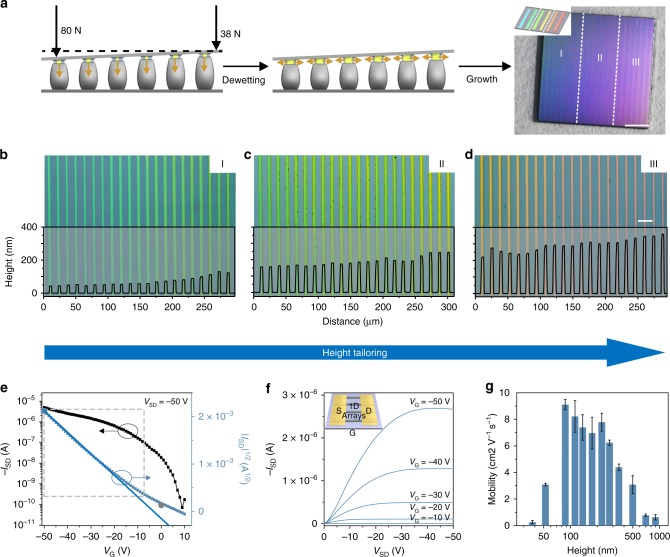
Height tailoring of ultrathin 1D organic nanobelt arrays. **a** Nucleation (left) and growth (middle) of organic crystals in the nano-confined space under linear gradient pressures. Digital photo showing the as-fabricated ultrathin nanobelt arrays with different sizes (divided into three areas as I, II, and III; right). **b**–**d** Typical optical micrographs of 1D arrays in the corresponding areas of I, II and III. Typical **e** output curve and **f** transfer curve of the device based on 1D arrays. The inset of **f** is the scheme of 1D array device (S: source electrode, D: drain electrode, and G: gate electrode). For the calculation of carrier mobility, the data in gray dash were employed, and the nattier blue and dark gray dots are marked as $\left| {I_{{\mathrm{DS}}}} \right|^{\max }$ $\left( {\left| {V_{\mathrm{G}}} \right|^{\max }} \right)$ and $I_{{\mathrm{DS}}}^0$, respectively. **g** The distribution diagram of mobility corresponding with the height of belts. The columns in the diagram from left to right refer to the arithmetical mean of the mobility measured by ultrathin organic belts with the height range of <50, 50–75, 75–100, 100–125, 150–200, 200–250, 250–300, 300–400, 400–600, 600–800, and ≥800 nm. Scale bars: **a** 1 mm, **d** 20 μm

For other patterning solution processes like dip-pen nanolithography and nanoimprinting lithography, a precisely leveled contact between the template and substrate is required to guarantee the solution diffusion control and pattern size control. In stark contrast, our assembly strategy possesses the asymmetric contact with a small inclination angle, then yielding the further height tailoring of ultrathin 1D arrays with a size gradient. We employed a pair of asymmetric pressures with 80 and 38 N onto the assembling system with the organic solution concentration of 2.5 mg mL^−1^ (Fig. 4a, left). After the generation of capillary bridges, the organic crystals nucleate and grow in both horizontal and vertical directions, causing the similar deformation of each micropillar in the whole template. While the continuous dewetting of organic solution follows the evaporation of solvents, the vertical strain and stress of each micropillar are improved with the vertical growth of crystals. Due to the different volume of each discrete confined space induced by the asymmetric pressures, gradient of crystal size in the confined space from left to right side (Fig. 4a, middle). The total evaporation of solvents guarantees the organic crystal growth, then gaining ultrathin 1D arrays with a height gradient (Fig. 4a, right). As shown in Fig. 4b, bright-field micrographs manifest nanobelts located in three regions (labeled in Fig. 4a, inset) with different colors complied with a spectrographic gradient from blue to red, indicating the gradually increased height of these nanobelts. Confocal laser scanning microscopy validated by AFM measurements (Supplementary Fig. 14) illustrates the heights ranging from *ca*. 50 to 350 nm (Fig. 4b–d). The statistics of nanobelt height in the three regions are plotted in Supplementary Fig. 15, verifying tunable height of 1D array in a large area. We further demonstrated the height tailoring of ultrathin 1D organic nanobelt arrays along the direction of 1D arrays (Supplementary Fig. 16a). During the crystallization in the asymmetric nano-confined capillary bridges, the evaporation at the side of low pressure is faster than the one at the other side due to their large exposure sectional area. This evaporation difference could cause the directional capillary flows from the high pressure side to the other side, then leading to the volumn gradient in every individual capillary bridges. As shown in Supplementary Fig. 16b–d, bright-field micrographs manifest nanobelts with the spectrographic gradient along the direction of 1D arrays from red to green, indicating the gradually decreased height of these nanobelts. We also used the V-type asymmetric pressure of 38, 80, and 38 N onto the assembly system by employing the PDMS substrate. We demonstrated the height tailoring of ultrathin 1D organic nanobelt arrays with a V-type tunable thickness varying from ca. 250 to 50 then to 250 nm (Supplementary Fig. 17).

### OFET device characterization

To demonstrate the applications of organic nanobelts as functional devices, organic field-effect transistors (OFETs) were constructed (details shown in Supplementary Note 1 and Note 2). A typical 1D array device presents the transfer and output characteristics with an on/off ratio of 2.5 × 10^4^, a preliminary mobility of 7.20 cm^2^ V^−1^ s^−1^ and an effective mobility of 4.51 cm^2^ V^−1^ s^−1^ as shown in Fig. 4e, f. Compared with nanobelts with the eliminated grain boundary and aligned crystallographic orientation, thin films with a p-channel (40 µm in width and 10 µm in length; schemed in Supplementary Fig. 18a) exhibits much lower electrical performance with a similar on/off ratio of 2.1 × 10^4^ but a low mobility of 0.0616 cm^2^ V^−1^ s^−1^ and effective carrier mobility of 0.0580 cm^2^ V^−1^ s^−1^ (Supplementary Fig. 18b, c). The average mobility of 1D array and thin film devices are 8.87 (±0.975) and 0.0590 (±0.0143) cm^2^ V^−1^ s^−1^, respectively, which are illustrated in Supplementary Fig. 18d. Average mobilities of ultrathin nanobelt arrays with different thickness were further measured (average mobility of 5 devices for each point, Fig. 4g).

### Morphology tailoring and generality

**Fig. 5:**
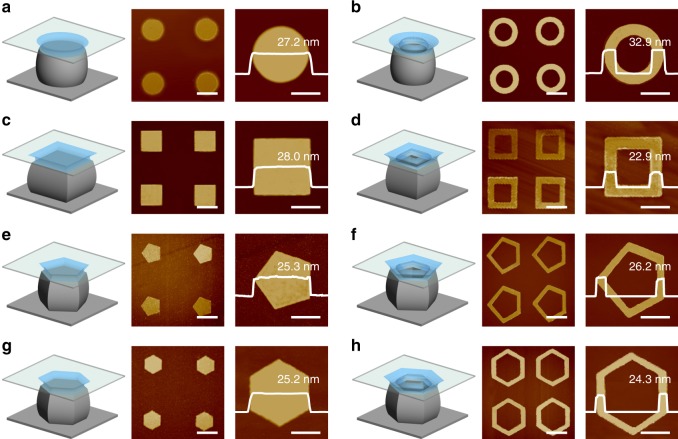
Morphology tailoring of single-crystalline organic ultrathin nanostructured arrays with tunable shapes. **a**–**h** Controlling assembly induced by confined crystallization of single-crystalline organic ultrathin nanostructured arrays with shapes of **a** circle, **b** circular ring, **c** square, **d** four-sided ring, **e** pentagon, **f** five-sided ring, **g** hexagon, and **h** six-sided ring using corresponding micropillars. Scale bars: **a**–**h** 20 μm (middle), 10 μm (right)

The versatility on confining the crystallization of single-crystalline organic ultrathin nanostructured arrays with different shapes is demonstrated through tailoring the geometry of micropillar templates. As show in Fig. 5, single-crystalline organic ultrathin nanostructured arrays with shapes of the circle, circular ring, square, four-sided ring, pentagon, five-sided ring, hexagon, and six-sided ring are assembled by the confined crystallization, which have repeatable thickness down to *ca*. 20 nm and high-quality morphology with sharp edge and smooth surface. These ultrathin nanostructured arrays are fabricated by employing corresponding micropillars.

To demonstrate the generality of this method, we fabricated ultrathin nanobelt arrays by using four representative classes of solution-processable materials, including organic small molecules, polymers, metal-halide perovskites and colloidal nanocrystals (details shown in the Supplementary information, Supplementary Note 3). Firstly, ultrathin nanobelt arrays with tunable thickness ranging from 10 nm to *ca*. 1 μm were successfully fabricated on two representative organic small molecules, 1,4-dimethoxy-2,5-di[4-(methylthio)styryl]benzene (TDSB) and C_8_-BTBT (Supplementary Figs. 19, 20). Secondly, we fabricated ultrathin 1D PBDT-STT polymer nanoarrays with the minimum thickness of 9 molecular layers (Supplementary Fig. 21). Ultrathin polymer arrays also present an edge-on stacking of polymer chains with the long-range order. Thirdly, (n-C_4_H_9_NH_3_)_2_(CH_3_NH_3_)_2_Pb_3_I_10_ was employed to construct perovskite single-crystal nanobelt arrays with highly ordered crystallographic orientation and tunable thickness ranging from 12 layers to *ca*. 1.5 μm (Supplementary Fig. 22). Fourthly, we demonstrated the fabrication of ultrathin 1D BaTiO_3_ nanocubes superlattice arrays with the minimum thickness of 19.1 nm, which is corresponding to 2 layers of nanocrystals (Supplementary Fig. 23). Through fabricating the ultrathin organic nanostructure arrays onto a series of flat substrates, including glass, silicon (Si) wafer, SiO_2_/Si wafer, polyimide (PI) and PDMS, we also demonstrated the general application of typical flat substrates in our method (Supplementary Fig. 24).

## Discussion

In summary, a solution method with controlled crystallization in a tunable nano-confined space has been invented to grow long-range-ordered solution-processable material arrays with pure crystallographic orientation, controlled alignment and tunable size. Through regulating the solution concentration and applied pressure, we demonstrated the height tailoring of belt arrays, which underlies the controlled nucleation rate and growth direction of TIPS-pentacene in the nano-confined space. In addition, we fabricated the ultrathin nanobelts with continuously tunable heights from less than 10 nm to *ca*. 1 μm, and also constructed large-area 1D arrays with a height gradient by employing asymmetric pressures. We can also control the assembly of organic ultrathin nanostructured arrays with different sizes and shapes. We suppose this versatile technique is also beneficial for the construction of heterojunction devices in the integrated circuit based on ultrathin nanostructures. This method opens up an opportunity to pattern designable and tunable ultrathin organic architectures with prominent optoelectronic performances for organic integration.

## Methods

### Fabrication of the photoresist micropillar template

Photolithography on the Si substrates (single side polishing) was performed by a mask aligner (ABM/6/350/NUV/DCCD/M) using SU-8 photoresist (MicroChem 2015) according to following steps. First, SU-8 photoresist was spin-coated on the polished surface of Si substrate at 6000 rpm for 60 s, then prebaked at 95 °C for 3.5 min. Second, photolithography on the SU-8 photoresist was performed by exposing the substrate under UV irradiation (the irradiance density is 15 mW cm^−2^) for 5 s with a mask. After postbaking at 95 °C for 4 min, the photoresist was developed in the developer for 60 s and rinsed in water for 5 s, then obtaining the photoresist micropillars. Finally, the pattern SU-8 photoresist was cured by hard baking for 15 min at 150 °C and exposing it under UV irradiation for its full crosslinking, then yielding stable 1D photoresist micropillars with high mechanical strength and high swelling resistance.

### Assembly of 1D single-crystal TIPS-pentacene arrays

The organic small molecule, TIPS-pentacene, was purchased from Sigma Aldrich without further purification. Then, the organic powders were dissolved in toluene (analytical reagent) to prepare the precursor solution. The photoresist template with micropillars was employed for guiding the dewetting of TIPS-pentacene solution. Ten microliter TIPS-pentacene solution was dropped onto the photoresist template and covered by the target substrate, yielding a sandwich-type system for the crystal growth. The integrated systems were heated at 80 °C to facilitate the solvent evaporation. Preparations of other solution-processable materials are shown in Supplementary Information (Part 6).

### Fabrication of TIPS-pentacene thin films

The TIPS-pentacene thin films were prepared by spin-coating method. The concentration of toluene solution was 2.5 mg mL^−1^, the film was fabricated at 2500 rpm for 30 s at room temperature, and then annealed at 100 °C for 5 min.

### Mechanical testing and FEA numerical modeling

We calculated the distribution of strain, stress and displacement of an individual pillars in the template with the height of 12 μm, width of 5 μm and gap of 8 μm. A series of pressures are gained by the equivalent tensile force of the pressure control system measured by an electronic tensile testing machine at different positions for the experimental analysis and mechanical simulation (ABAQUS FEA software package). Owing to the periodic arrangement of the micropillar template, the simulation based on one period is sufficient. The whole system consists of SU-8 photoresist micropillars, silicon flat and glass or silicon substrate, which were all treated as linear elastic materials. The Young’s modulus and Poisson’s ratios of these materials are 2 GPa, 0.33, 190 GPa, 0.28, 72 GPa, 0.2 (or 190 GPa, 0.28), respectively. The plane strain element CPE4R was adopted for all the materials due to the uniform pressure along with the vertical direction induced by the negligible length-width and length-height ratio of micropillars. In addition, the establishment of simulation is based on two assumptions: (i) photoresist micropillars is solidly bonding on the Si flat, and (ii) the slippage is free between the top of micropillars and glass or silicon substrate with the lubrication of organic solution. The buckling simulation is realized by the linear perturbation analysis.

### Characterization

Digital photograph of the sample with height tailoring is achieved by a digital camera (Canon, 80D, Japan). The microscope images of all the aligned microcrystal arrays were acquired by an optical microscope (Vision Engineering Co., UK), which was coupled to a charge-coupled device (CCD) camera. The polarizing characteristics were acquired through polarizing microscope (Olympus, BX51, Japan). Static contact angles were measured on a Dataphysics OCA20 contact angle system (Germany) at room temperature. The average contact angle was obtained by measuring more than five different positions of the same sample. The thickness of large-area 1D arrays with a height gradient was measured by a confocal laser scanning microscope (Olympus, OLS-4500, Japan). SEM (JEOL, JSM-7500F, Japan) was used to investigate the structure of the photoresist pillar template and the as-prepared microribbon arrays at an accelerating voltage of 5.0 kV. Cross-sectional view images of the template and assembly system are observed by an ESEM (FEI, QUANTA FEG 250, USA). The morphological and crystalline characteristics were further investigated by TEM (JEOL, JEM-2100, Japan) operating at a 200 kV accelerating voltage. AFM measurements of the morphology of belts and thin film were carried out with a Nanoscope IIIa instrument (Bruker, ICON2-SYS, Germany). A powder X-ray diffractometer (Bruker, D8 focus, Germany) is used for evaluating crystal structures with monochromatized Cu K_α_ radiation (λ = 1.54 Å). GIWAXS is performed on XEUSS SAXS/WAXS system with a incidence angle of 0.2° to certify the crystallization property, the distance between sample and detector is 122 mm.

## Supplementary information

Supplementary Information

## Data Availability

The data that support the findings of this study are available from the corresponding author upon reasonable request.
