# Supplementary material for: Nano-confined crystallization of organic ultrathin nanostructure arrays with programmable geometries
Source: Nat Commun. 2019 Sep 2;10:3912. doi: 10.1038/s41467-019-11883-6 (PMC6718603; doi:10.1038/s41467-019-11883-6)
Supplement: Supplementary file 1 — Supplementary Information [file 41467_2019_11883_MOESM1_ESM.pdf]

Supplementary information

Nano-confined Crystallization of Organic Ultrathin Nanostructure Arrays with Programmable Geometries

Gao, et al.

Part 1. Fabrication and characterization of photoresist-pillar templates

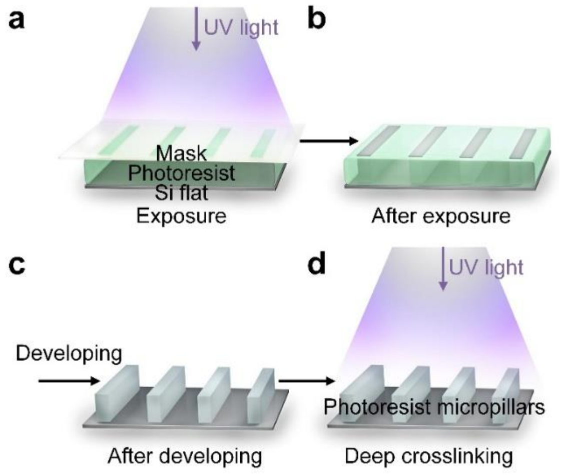

Supplementary Fig. 1 | Fabrication of the photoresist-pillar templates. a The as-prepared spin-coated photoresist film on the silicon flat exposed under ultraviolet light with covered by photomask. **b** The exposed parts with cross-linked after exposure and removing the mask. **c** 1D photoresist micropillars achieved by removing redundant parts without crosslinking. **d** Employing ultraviolet illumination again to deepen the cross-linking between long polymer molecular chains to realize a stable template.

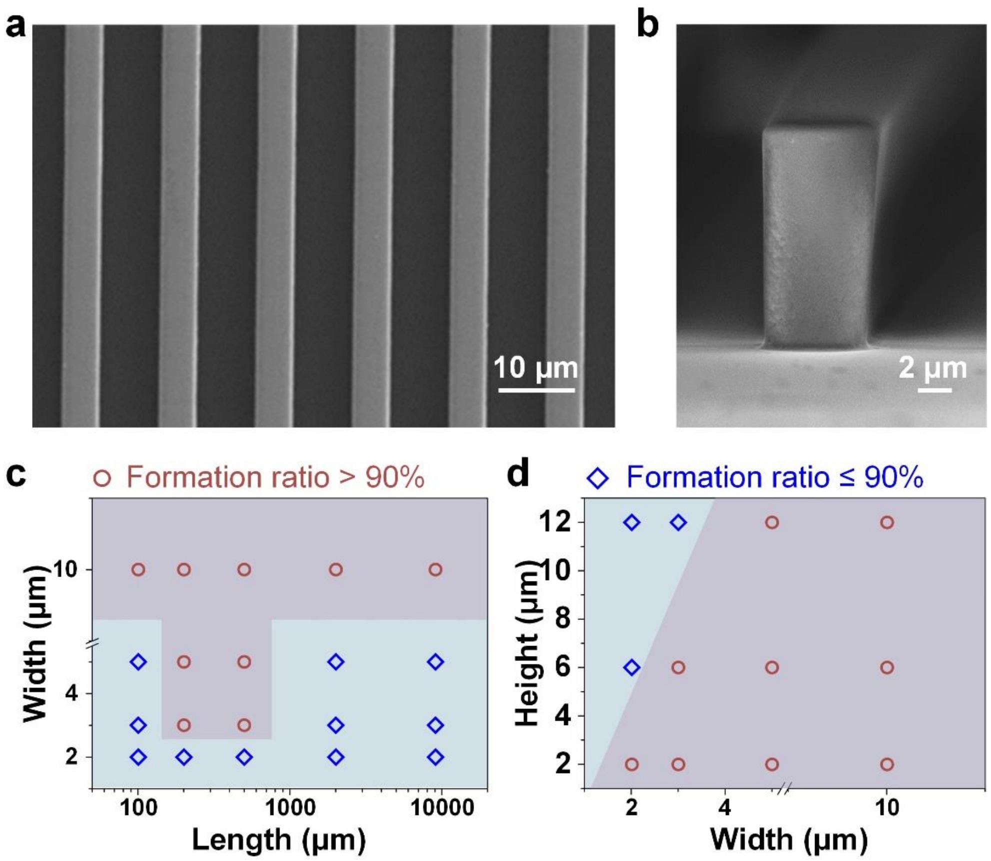

Supplementary Fig. 2 | Size and formation ratio of 1D micro-structured photoresist pillar templates. **a** SEM image of template with the width of 5 μm and gap of 8 μm. **b** Zoom-in SEM with bird's-eye view of an individual micropillar with the width of 5 μm and height of 12 μm in **a**. The statistical formation ratios of photoresist micropillars with different **c** width/length and **d** height/width ratios (statistical formation ratio of 10 samples for each point, red circles in the violet background mean the high formation ratio (>90%) of photoresist micropillars, blue diamonds in the cyan background mean the low formation ratio (≤90%) of photoresist micropillars).

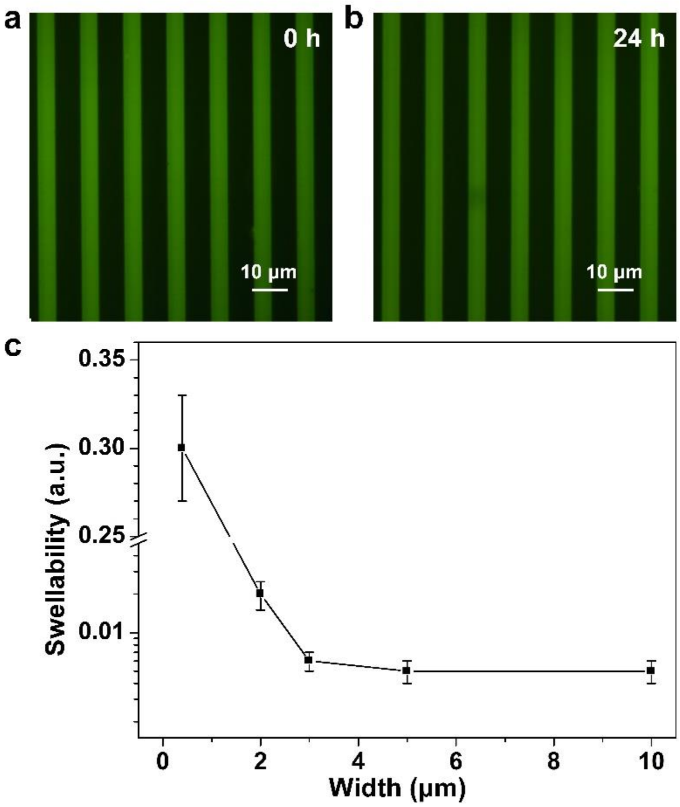

Supplementary Fig. 3 | Swelling resistance of photoresist micropillar template. Fluorescence microscopy images of photoresist micropillars **a** before immersion and **b** with immersed in toluene for 24 h. **c** The statistical swelling ratios of photoresist micropillars with different widths (average height of 5 micropillars for each point).

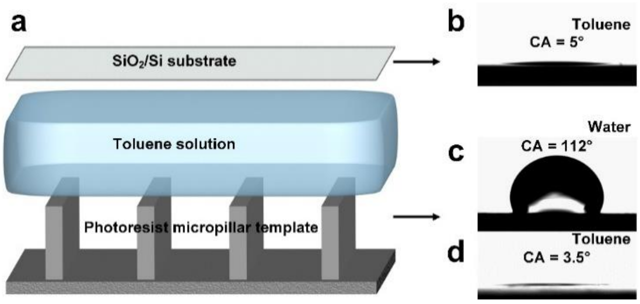

Supplementary Fig. 4 | The assembly system and its surface wettability. a Schematic illustration of the sandwich-type assembly system composed of SiO_2/Si substrate, organic solution and photoresist micropillar template. **b** The static contact angle of toluene is 5 °, showing the superlyophilicity of the substrate with lyophilic treatment. The static contact angle of **c** water droplet and **d** toluene are respectively 112 ° and 3.5 °, indicating the hydrophobicity and superoleophilicity of the template, which is the consequence of the nonpolar alkyl side chain of SU-8 molecular and smooth surface of the pillars.

Part 2. Mechanical simulation and observation of the assembly system under different pressures

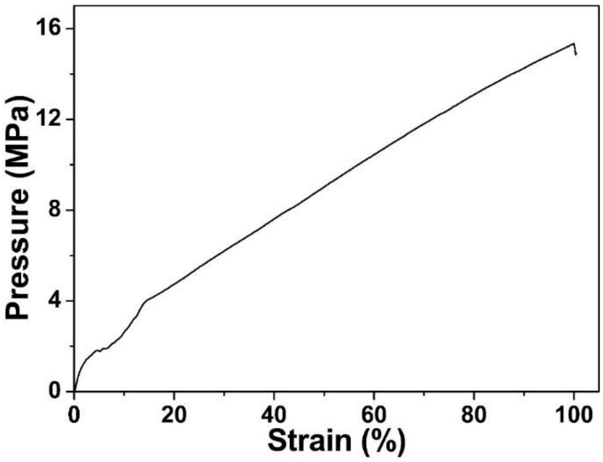

Supplementary Fig. 5 | The strength test of the pressure system through an electronic tensile machine. An almost linear curve plotted in the pressure-strain diagram indicates the linear strain response of the assembling system with tuning pressure.

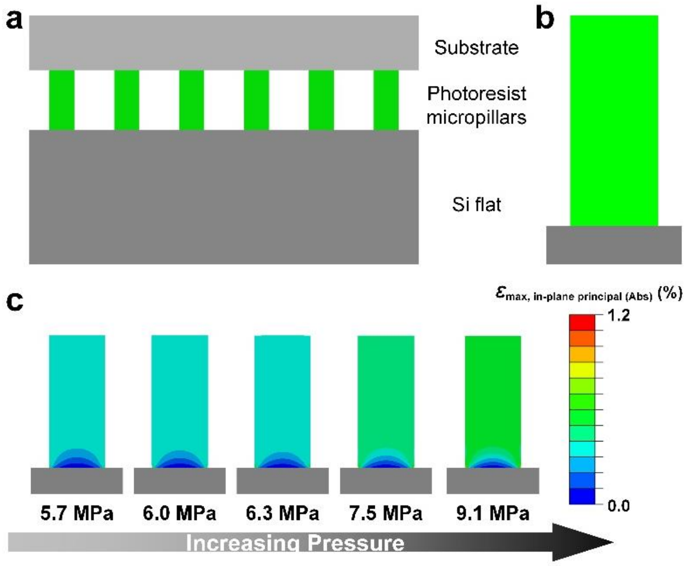

Supplementary Fig. 6 | Mechanical simulation model of the assembly system. a The mechanical simulation model of the micropillar template in the assembly system established by FEA with the gap of 8 μm. **b** Initial state of an individual micropillar with the width of 5 μm and the height of 12 μm in the simulation model. **c** Simulated strain distribution along the vertical direction of a typical photoresist micropillar, showing a diminution of in-plane maximum principal strain, ε_{max}, with pressure increasing from 5.7 to 9.1 MPa.

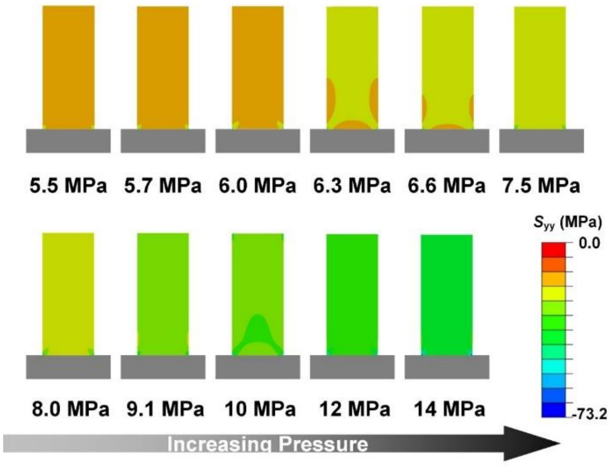

Supplementary Fig. 7 | Simulated stress distribution of photoresist micropillars under different pressures. Simulated stress distribution along the vertical direction of a typical photoresist micropillar, showing a diminution of the stress (absolute values) along the height direction of micropillars, S_{yy}, with pressure increasing from 5.5 to 14 MPa.

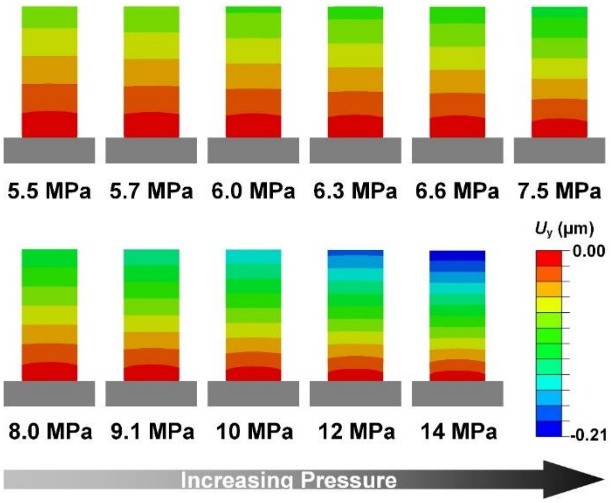

Supplementary Fig. 8 | Simulated displacement distribution of photoresist micropillars under different pressures. Simulated displacement distribution along the vertical direction of a typical photoresist micropillar, showing a diminution of the displacement (absolute values) along the height direction of micropillars, U_y, with pressure increasing from 5.5 to 14 MPa.

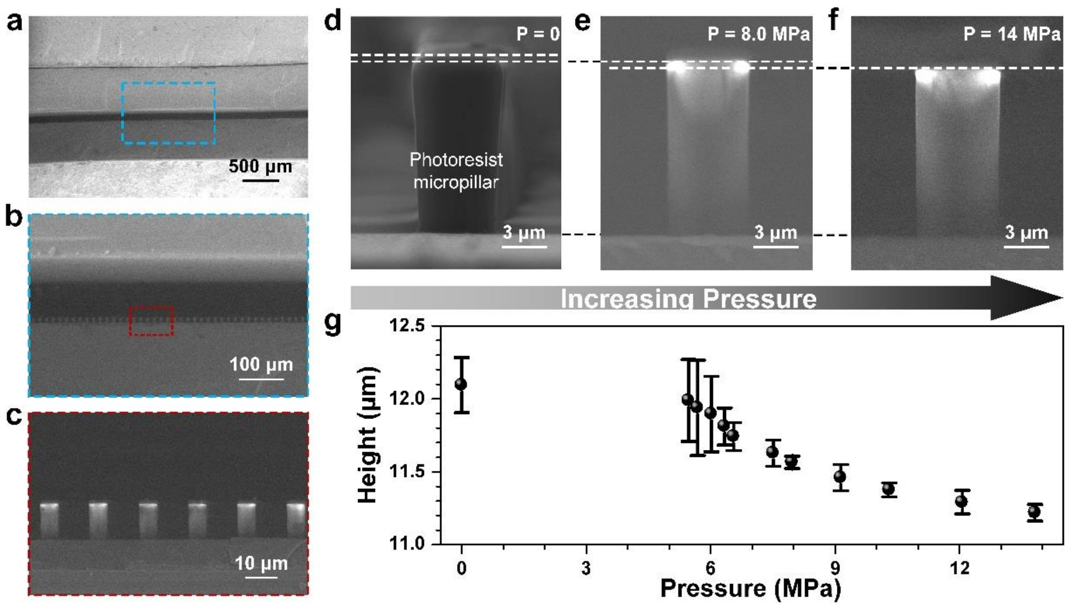

Supplementary Fig. 9 | The cross-sectional view observation of the sandwich-type assembly system and its deformation through an individual micropillar with increasing pressure from 0 to 14 MPa. a Cross-section SEM image of the whole assembly system, illustrating the glass roof, silicon substrate, photoresist micropillar template and glass baseboard from top to bottom. **b** Zoom-in SEM image of the system in blue wireframe of **a**. **c** Zoom-in SEM image of the system in red wireframe of **b**. **d-f** Cross-sectional view ESEM images of an individual micropillar under different pressure of 0, 8.0 and 14 MPa, respectively, evidencing a nearly linear strain with observable longitudinal shrinkage marked by the white dashed line. **g** The statistical diagram of the micropillars‘ height under different pressure, showing the definite trend of micropillar deformation (average height of 5 micropillars for each point).

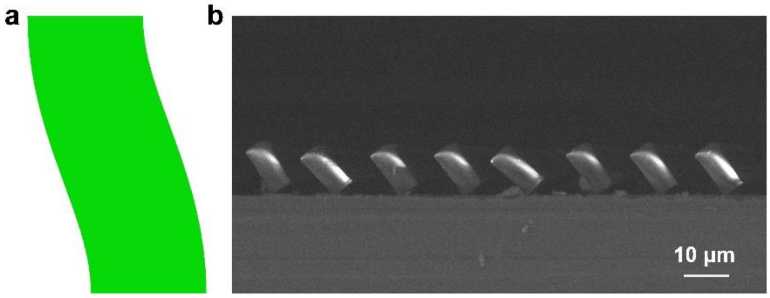

Supplementary Fig. 10 | Simulated and experimental results of destroyed micropillars. a The first-order buckling mode of an individual micropillar simulated by FEA (the critical pressure is 39 MPa). **b** ESEM image of fractured micropillars under overlarge pressure beyond the critical value.

Part 3. Morphology and crystallography of organic ultrathin nanobelt arrays compared with spin-coated films

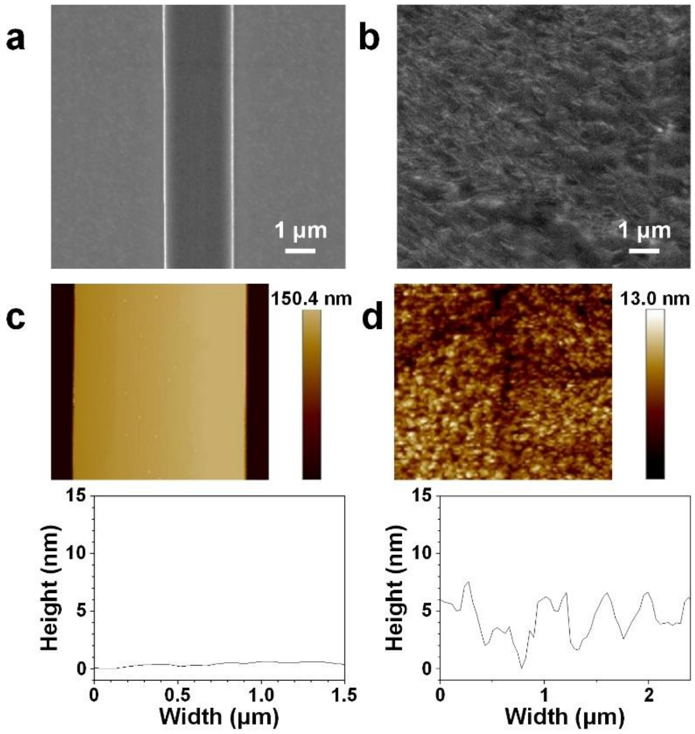

Supplementary Fig. 11 | Morphology of TIPS-pentacene single-crystalline nanobelts and polycrystalline film. SEM images of **a** an individual organic nanobelt and **b** spin-coating film. AFM images of **c** an individual nanobelt and **d** film corresponding with the root mean square roughness (R_q) of 0.416 and 3.26 nm, respectively.

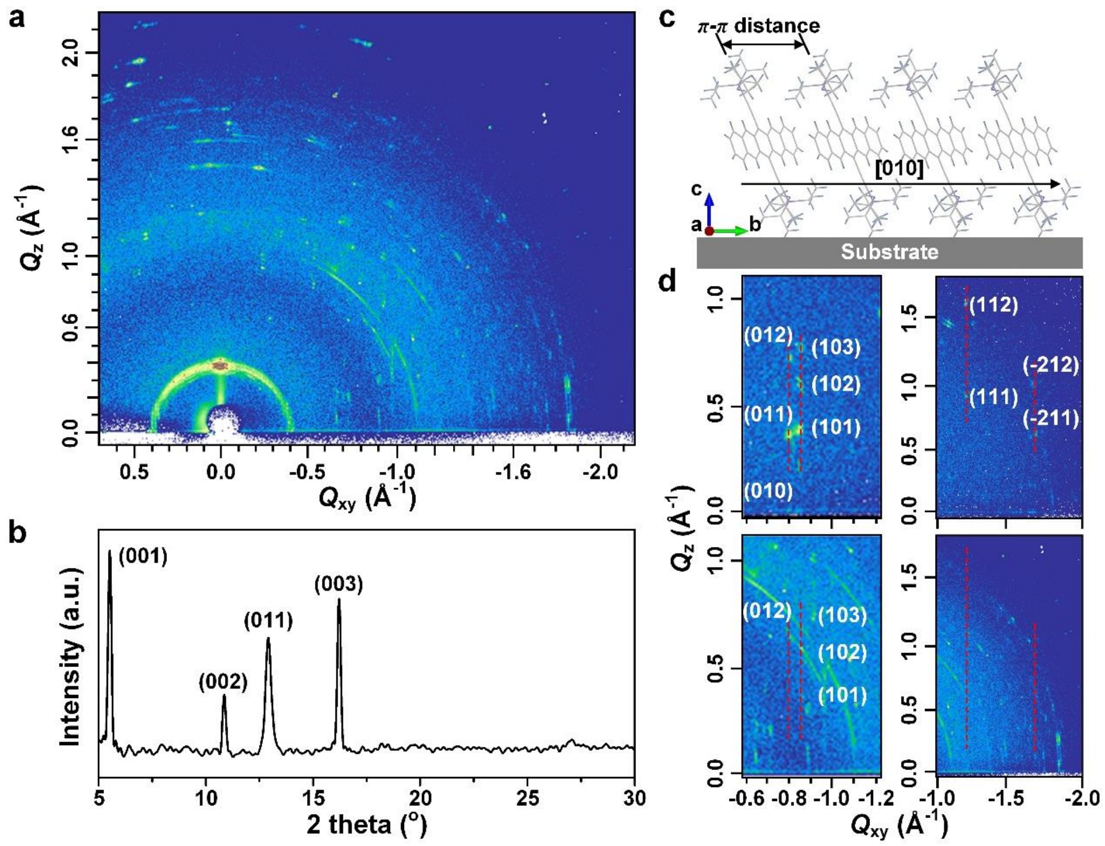

Supplementary Fig. 12 | Crystallography of TIPS-pentacene film on the silicon substrate and a crystallographic comparison of film and 1D arrays. a GIWAXS image and **b** XRD diagram of the organic film fabricated by spin-coating method. **c** Schematic illustration of molecule packing and crystal orientation of 1D TIPS-pentacene arrays on the silicon substrate, showing the growing direction of the 1D single crystals along with crystal orientation of [010]. This directional growth is consistent with the *π*-*π* stacking direction and benefits to carrier transport. **d** Zoom-in GIWAXS patterns of 1D arrays (up) and thin film (down) with comparative analysis.

Part 4. Size tailoring of organic ultrathin nanobelt arrays with different heights and widths

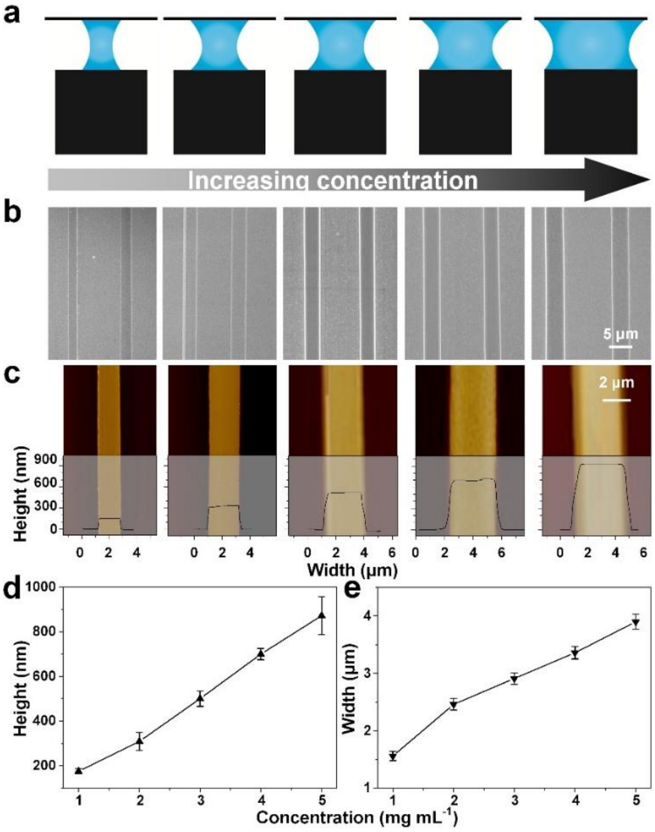

Supplementary Fig. 13 | Mechanism and statistical results of size changing with the control of solution concentration under a static pressure of 5.5 MPa. a Schematic diagram of changing capillary bridge corresponding with concentration, showing an enlarged capillary bridge with the concentration increasing. **b** SEM and **c** AFM images of as-fabricated 1D arrays with concentration increasing, showing the growing width and height of nanobelts with the concentration increasing. The statistical charts of the **d** height and **e** width corresponding with concentration increasing, respectively, manifesting the positive correlation between the solution concentration and size in three dimensions of organic nanobelts (average height and width of 5 belts for each point).

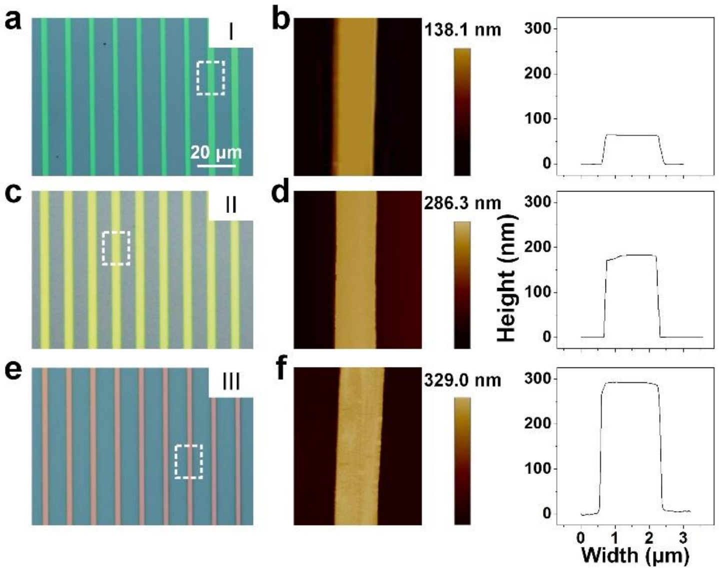

Supplementary Fig. 14 | Height tailoring of 1D organic arrays with verification. Zoom-in optical micrographs of 1D arrays in the three areas of **a** I, **c** II and **e** III, respectively. **b**, **d** and **f** AFM images and diagrams of the nanobelts in the white wireframe of **a**, **c** and **e**, respectively.

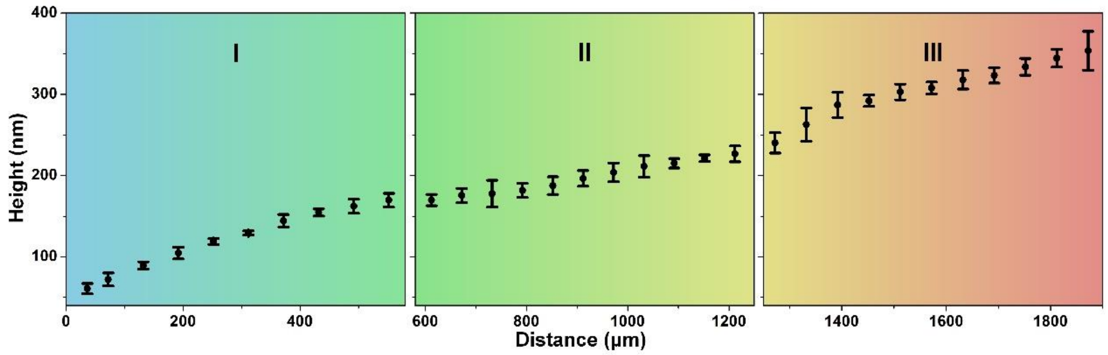

Supplementary Fig. 15 | Height tailoring of organic belt arrays. Statistical results of nanobelt height increasing from ca. 60 to 350 nm with loading gradient pressure (average height of 3 belts for each point).

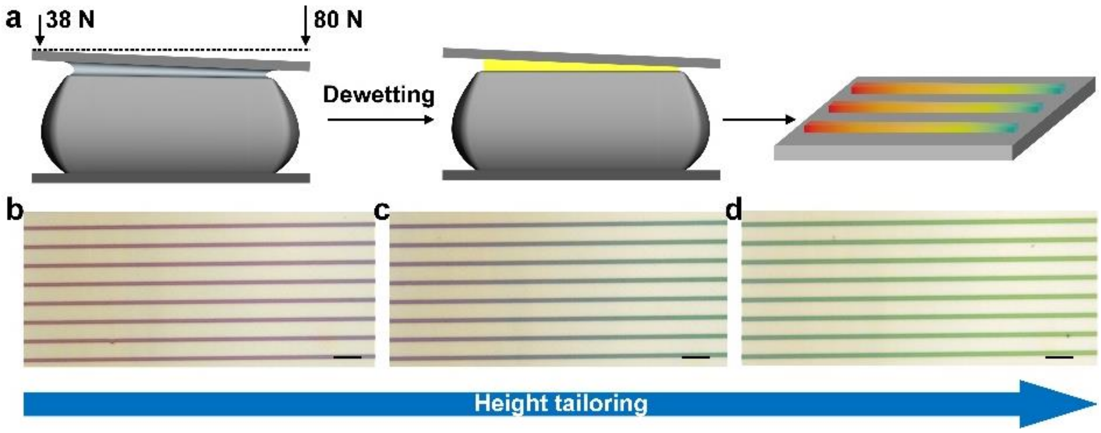

Supplementary Fig. 16 | Height tailoring of ultrathin 1D organic belt arrays along the direction of 1D arrays. a Schematic illustration of the dewetting and crystallization. **b-d** Typical optical micrographs of 1D arrays in different areas. Scale bar: 20 μm.

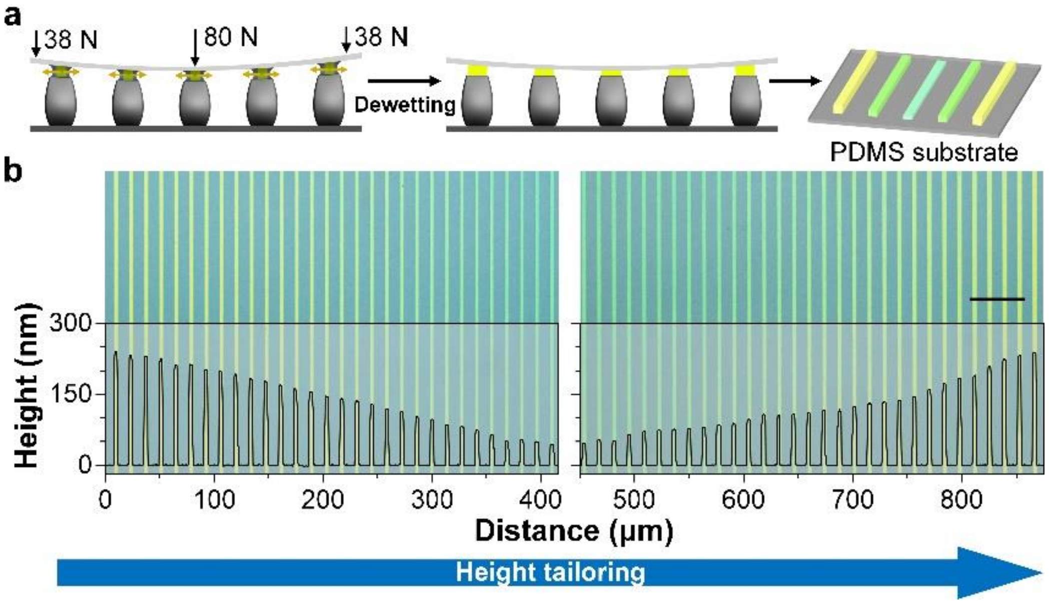

Supplementary Fig. 17 | Height tailoring of ultrathin 1D organic nanobelt arrays with a "V"-type tunable thickness on the PDMS substrate. a Schematic illustration of the dewetting and crystallization. **b** Typical optical micrographs of 1D arrays in different areas. Scale bar: 50 μm.

Part 5. Characterization of OFET devices based on organic ultrathin nanobelt arrays with different heights and widths compared with spin-coated films

Supplementary Note 1. Details on OFET device performances of organic ultrathin nanobelt arrays with different heights and widths and spin-coated films

To demonstrate the applications of organic nanobelts as functional devices, OFETs were constructed (schematically illustrated in Fig. 4f, inset). A typical 1D array device with channel width of 6.0 μm 1.0 μm and channel length of 15 μm presents the transfer and output characteristics as shown in Fig. 4e, f. From the transfer curve, the on/off ratio is measured as 2.5×10^4. The mobility of device was calculated by considering the ideal linear FET behavior and introducing a reliability factor (*r*), which was suggested by Choi et al[1]. The transfer curve of our devices approximately coincides with the ideal linear FET model, yielding a preliminary mobility of 7.20 $cm^2\ V^{-1}\ s^{-1}$ and an effective mobility of 4.51 $cm^2\ V^{-1}\ s^{-1}$ after including the *r*.

Compared with nanobelts with the eliminated grain boundary and aligned crystallographic orientation, the thin films with rough surface and disordered orientation (schematically illustrated in Supplementary Fig. 16a exhibits much lower electrical performance. From the typical transfer and output curves, OFET devices based on TIPS-pentacene thin film with channel width of 40 μm and channel length of 10 μm perform a similar on/off ratio of 2.1×10^4 but a low mobility of 0.0616 $cm^2\ V^{-1}\ s^{-1}$ with the reliability factor of 0.941 and effective carrier mobility of 0.0580 $cm^2\ V^{-1}\ s^{-1}$ (Supplementary Fig. 16b, c). The average mobility of 1D array and thin film devices are 8.87 (±0.975) and 0.0590 (±0.0143) $cm^2\ V^{-1}\ s^{-1}$, respectively, which are illustrated in Supplementary Fig. 16d.

To further evaluate the ultrathin 1D arrays OFETs, the average mobilities of ultrathin nanobelt arrays with different thickness were measured (Fig. 4g), then demonstrating an interesting variation trend of their electronic performances when tuning the thickness of nanobelts. For the devices based on the nanobelts with the thin range (<50 nm), they perform very low mobility of 0.260 (±0.129) $cm^2\ V^{-1}\ s^{-1}$ as similar as the performance of

thin film devices, perhaps caused by the imperfect surface morphology of ultrathin nanobelts resembling the spin-coating membrane. With the height of nanobelt increasing from 50 to 100 nm, the nanobelt arrays exhibits an enhanced mobility from 0.260 (±0.129) to 9.11 (±0.388) $cm^2 V^{-1} s^{-1}$ owing to the promoted carrier transport induced by improved quality of nanobelts. After the plateau region with the height ranging from 75 to 250 nm, the mobility of 1D arrays presents an attenuation from 7.80 (±0.678) to 0.624 (±0.203) $cm^2 V^{-1} s^{-1}$ with the boosted height more than 300 nm due to the poor contact between the organic semiconductor and the electrodes[2].

Supplementary Note 2. Additional experimental details on fabrication and characterization of OFET devices

Fabrication and characterization of OFET devices: Top-contact devices of the patterned small molecule nanobelt arrays were directly fabricated on OTS treated Si/SiO_2 (300 nm) substrates by evaporating gold film as source and drain electrodes without any annealing operation.

All electrical characteristics of the devices were measured in an ambient environment at room temperature. The OFET characteristics were measured through a Keithley 4200 SCS semiconductor parameter analyzer. The carrier mobility in the saturation regime at gate voltage (V_G) of -50 V could be calculated by

$$\mu = \frac{2L}{WC_i}\left(\frac{\partial\sqrt{|I_{DS}|}}{\partial V_G}\right)^2 \quad (1)$$

where μ is carrier mobility, L is defined as the channel length, W is the channel width, C_i is the capacitance of the gate dielectric layer per unit area and I_{DS} is the current between the drain and source electrodes. To evaluate the compliance of OFET behavior to the physical laws, the reliability factor of the measurement in the saturation regime is required to be achieved by

$$r_{sat} = \left(\frac{\sqrt{|I_{DS}|^{max}} - \sqrt{|I_{DS}^0|}}{|V_G|^{max}}\right)^2 \Bigg/ \left(\frac{WC_i}{2L}\mu_{sat}\right)_{experimental} = \left(\frac{\sqrt{|I_{DS}|^{max}} - \sqrt{|I_{DS}^0|}}{|V_G|^{max}}\right)^2 \Bigg/ \left(\frac{\partial\sqrt{|I_{DS}|}}{\partial V_G}\right)^2_{experimental} \quad (2)$$

where $|I_{DS}|^{max}$ denotes the experimental maximum drain-source current under the maximum gate voltage $|V_G|^{max}$ (nattier blue dot) and I_{DS}^0 is the drain-source current at gate voltage of 0V (dark grey dot) (Supplementary Fig. 17). Thus, the effective carrier mobility could be emendated by equation (3).

$$\mu_{eff} \equiv r_{sat} \times \mu_{experimental} \quad (3)$$

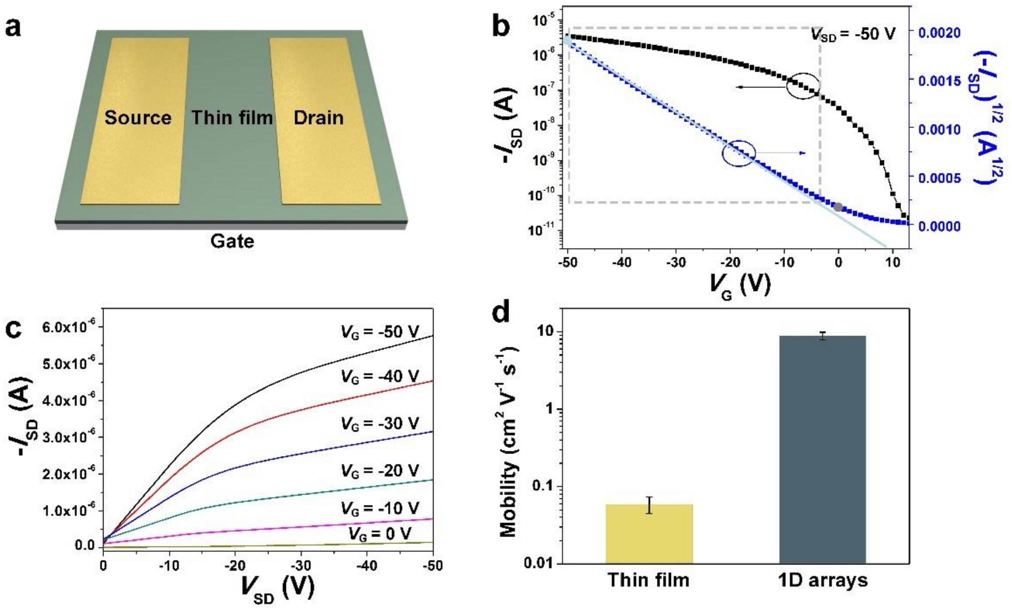

Supplementary Fig. 18 | Electronic performing of OFET devices based on thin film. **a** Schematic diagram of thin film OFET. Typical **b** output curve and **c** transfer curve of the device based on thin film device. For the calculation of carrier mobility, the data in gray dash of the transfer curves were employed, and the nattier blue and dark grey dots are marked as $\left|I_{\mathrm{DS}}\right|^{\max}$ ($\left|V_{\mathrm{G}}\right|^{\max}$) and I_{DS}^{0}, respectively. **d** The distribution diagram of carrier mobility achieved by the transfer curves of 1D array with the height from 75 to 300 nm and thin film devices (average mobility of 10 devices for each point).

Part 6. Height tailoring of organic ultrathin nanobelt arrays with different solution-processable materials

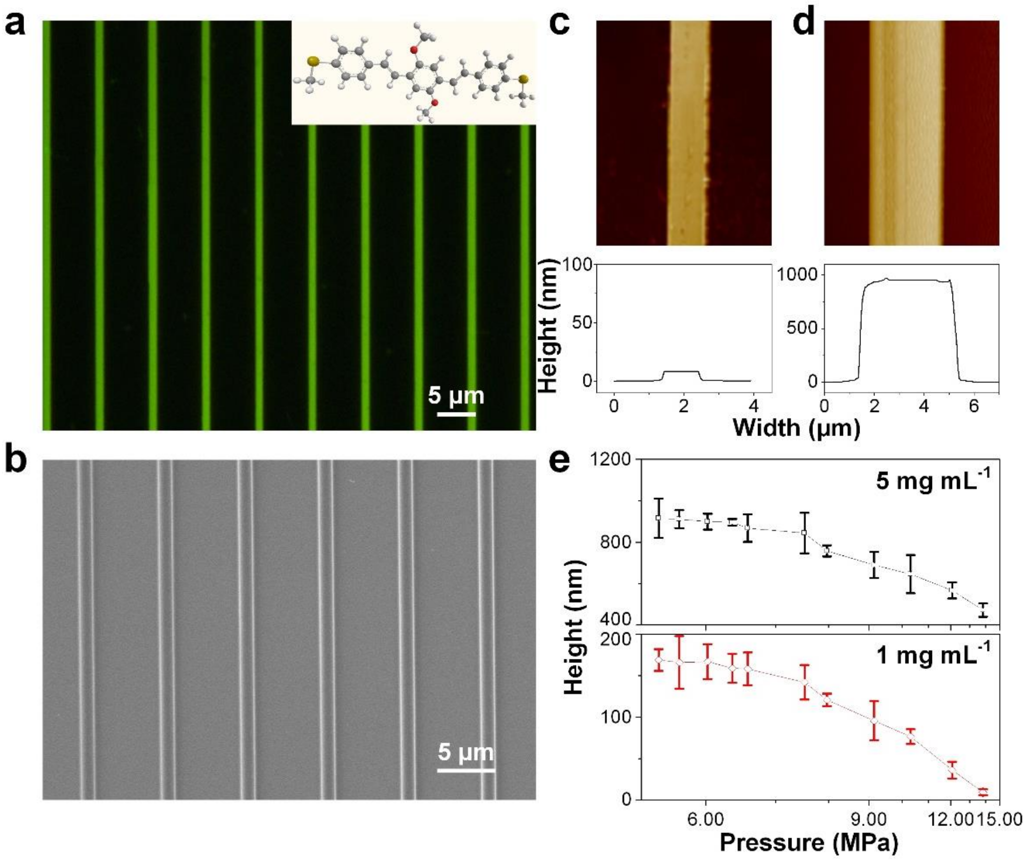

Supplementary Fig. 19 | Height tailoring of 1D TDSB organic arrays. a Fluorescence micrograph and **b** SEM image of 1D single-crystalline arrays, illustrating the high quality of TDSB nanobelts. AFM images of TDSB nanobelts with the height of **c** 10.3 nm and **d** 950 nm, which are fabricated at the low concentration of 1 mg mL^{-1} under the high pressure of 14 MPa and at 5 mg mL^{-1} under 5.5 MPa, respectively. **e** Pressure dependent height of TDSB nanobelts at solution concentrations of 1 and 5 mg mL^{-1}. The crystal structure of TDSB is reported as a monoclinic structure with a = 8.15 Å, b = 5.42 Å, c = 26.14 Å, $\alpha = \gamma = 90°$, $\beta = 97.37°$ and space group of $P2_1/c$[3] (average height of 5 belts for each point). Owing to the zone axe of [001] determined as perpendicular to the long axis of TDSB nanobelts, the minimum height of nanobelts (10.3 nm) is corresponding to 4 layers of moleclues. The organic small molecule, TDSB, was purchased from Sigma Aldrich without further purification.

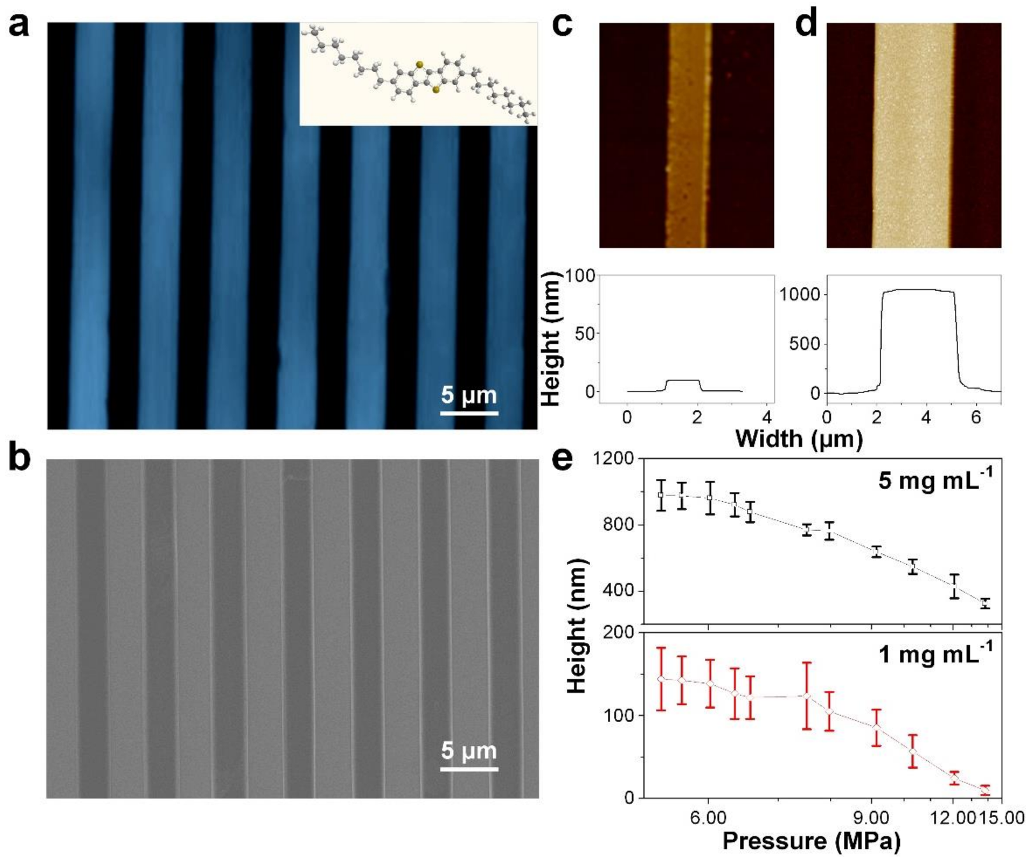

Supplementary Fig. 20 | Height tailoring of 1D C_8-BTBT organic arrays. a Fluorescence micrograph and **b** SEM image of 1D single-crystalline arrays, illustrating the high quality of C_8-BTBT nanobelts. AFM images of C_8-BTBT nanobelts with the height of **c** 9.7 nm and **d** 1050 nm, which are fabricated at the low concentration of 1 mg mL^{-1} under the high pressure of 14 MPa and at 5 mg mL^{-1} under 5.5 MPa, respectively. **e** Pressure dependent height of C_8-BTBT nanobelts at solution concentrations of 1 and 5 mg mL^{-1} (average height of 5 belts for each point). The crystal structure of C_8-BTBT is reported as a monoclinic structure with a = 6.35 Å, b = 8.56 Å, c = 24.47 Å, $\alpha = \gamma = 90°$, β = 92.443 ° and space group of $P2_1/a$[4]. Owing to the zone axe of [001] determined as perpendicular to the long axis of TDSB nanobelts, the minimum height of nanobelts (9.7 nm) is corresponding to 4 layers of moleclues. The organic small molecule, C_8-BTBT, was purchased from Sigma Aldrich without further purification.

Supplementary Note 3. Additional discussion on general application of ultrathin array fabrication with employing other solution-processable materials

To our knowledge, typical solution-processable optoelectronic materials include polymers, halide perovskites and nanoparticles besides organic small molecules. Thus, we employed PBDT-STT (polymer), $(BA)_2(MA)_2Pb_3I_{10}$ (perovskite), $BaTiO_3$ nanocubes (nanoparticle) for demonstrating the general applicability of our method. The polymer, PBDT-STT, was purchased from Solarmer Energy without further purification. Perovskite was synthesized by a previous report[5]. Nanocrystals were synthesized by a modified hydrothermal method[6].

For the polymer PBDT-STT, we demonstrated the fabrication of 1D polymer arrays with large area (approximately 300×200 μm^2), precise position, low misalignment angle (<3 °), smooth surface and straight boundary, ordered orientation (edge-on stacking) and tunable thickness (average thickness from ca. 23 nm to ca. 1.1 μm) (Supplementary Fig. 21). For the perovskite $(BA)_2(MA)_2Pb_3I_{10}$, we demonstrated the fabrication of 1D perovskite arrays with large area (approximately 300×200 μm^2), precise position, low misalignment angle (<3 °), smooth surface and straight boundary, ordered [010] orientation and tunable thickness (average thickness from *ca.* 20 nm to *ca.* 1.5 μm) (Supplementary Fig. 22). For the $BaTiO_3$ nanocubes, we demonstrated the fabrication of 1D assembled superlattice arrays with large area (approximately 300×200 μm^2), precise position, low misalignment angle (<1 °), smooth surface, straight boundary, and tunable thickness (average thickness from *ca.* 20 nm to *ca.* 1.1 μm) (Supplementary Fig. 23).

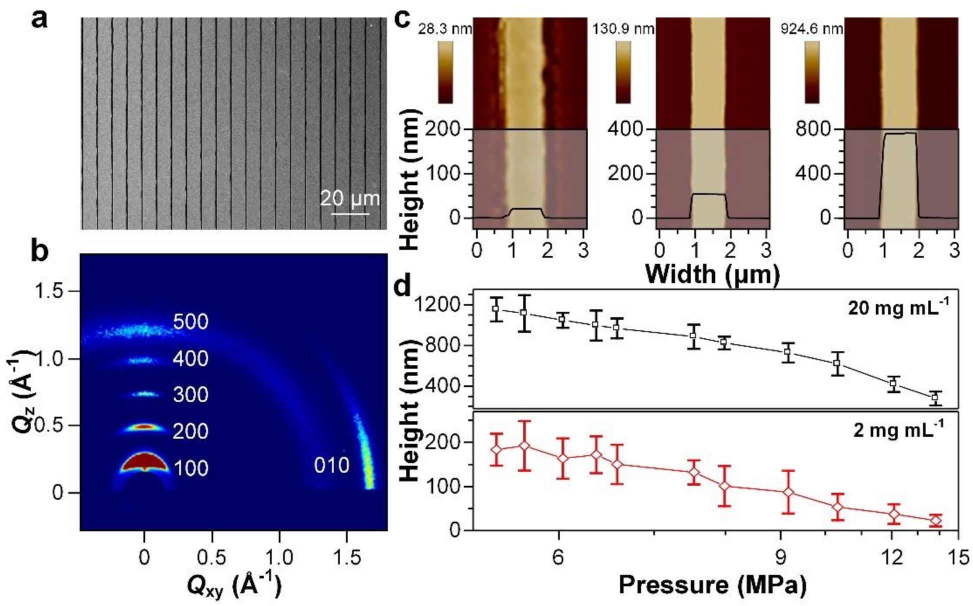

Supplementary Fig. 21 | Height tailoring of 1D PDBT-STT polymer arrays. a SEM image of 1D polymer arrays, illustrating the high quality of PBDT-STT nanobelts. **b** GIWAXS pattern of 1D polymer arrays, indicating the edge-on stacking of polymer chains. Typical AFM images of polymer nanobelts with the height of **c** 21.3 nm (left), 108.5 nm (middle) and 764.7 nm (right), which are fabricated at the concentration of 2 mg mL^{-1} under the high pressure of 14 MPa and low pressure of 5.5 MPa, and at 20 mg mL^{-1} under 8.0 MPa, respectively. **d** Pressure dependent height of PBDT-STT nanobelts at solution concentrations of 2 and 20 mg mL^{-1} (average height of 5 belts for each point).

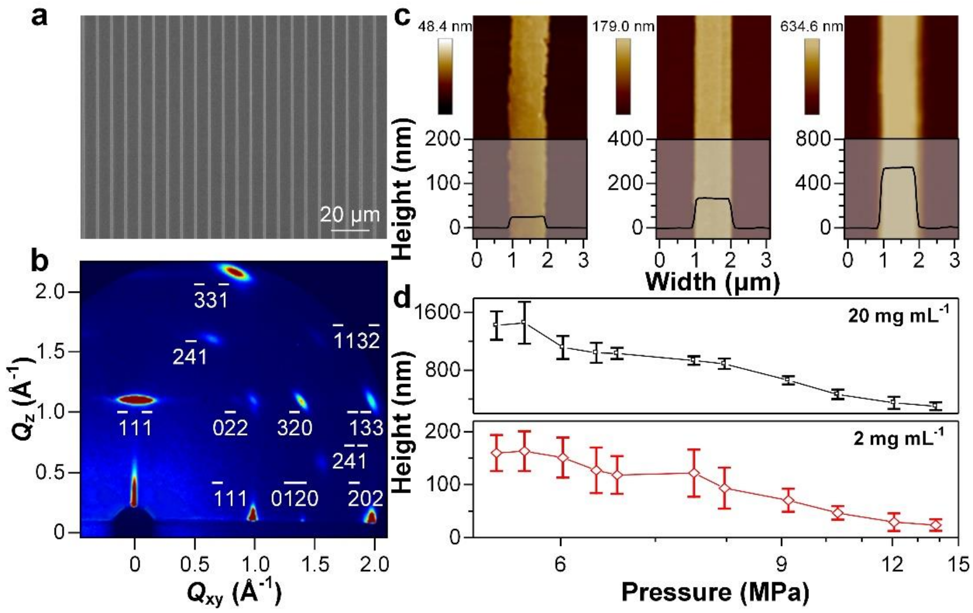

Supplementary Fig. 22 | Height tailoring of 1D $(BA)_2(MA)_2Pb_3I_{10}$ perovskite arrays. **a** SEM image of 1D perovskite arrays, illustrating the high quality of nanobelts. **b** GIWAXS pattern of 1D perovskite arrays, indicating the crystal orientation of 1D arrays along with [101]. Typical AFM images of perovskite nanobelts with the height of **c** 25.6 nm (left), 135.1 nm (middle) and 542.5 nm (right), which are fabricated at the low concentration of 2 mg mL^{-1} under the high pressure of 14 MPa and low pressure of 5.5 MPa, and at 20 mg mL^{-1} under 9.1 MPa, respectively. **d** Pressure dependent height of perovskite nanobelts at solution concentrations of 2 and 20 mg mL^{-1} (average height of 5 belts for each point). The crystal structure of $(BA)_2(MA)_2Pb_3I_{10}$ is reported as a monoclinic structure with a = 8.95 Å, b = 39.35 Å, c = 8.86 Å, $\alpha = \beta = \gamma$ = 90°, and space group of *Cmcm*. Owing to the zone axe of [101] determined as perpendicular to the long axis of perovskite nanobelts, the minimum height of nanobelts (23.8 nm) is corresponding to 18 layers of moleclues.

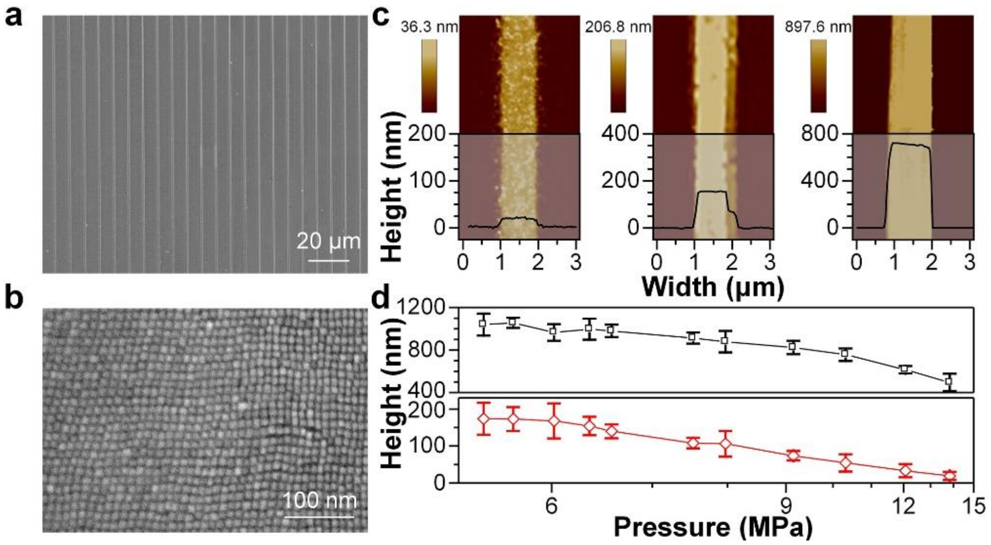

Supplementary Fig. 23 | Height tailoring of 1D arrays of $BaTiO_3$ nanocubes. a SEM image of 1D single-crystalline arrays. **b** Zoom-in SEM image of an individual 1D assembled nanostructure with high magnification, illustrating the superlattice of nanocubes. AFM images of 1D assembled nanostructure with the height of **c** 20.6 nm (left), 159.4 nm (middle) and 718.8 nm (right), which are fabricated at the low concentration of 1 mg mL^{-1} under the high pressure of 14 MPa and low pressure of 5.5 MPa, and at 5 mg mL^{-1} under 8.0 MPa, respectively. **d** Pressure dependent height of 1D assembled nanostructures at solution concentrations of 1 and 5 mg mL^{-1} (average height of 5 belts for each point). Owing to the size of nanocube with *ca.* 10 nm in length, the minimum height of 1D assembled nanostructures (19.1 nm) is corresponding to 2 layers of nanocubes.

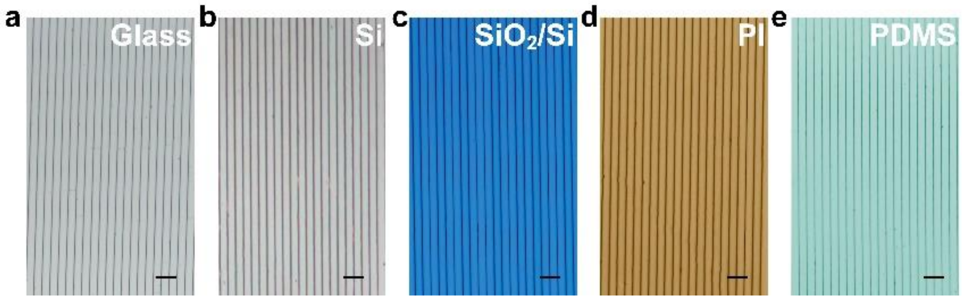

Supplementary Fig. 24 | Fabrications on different flat substrates. As-fabricated ultrathin polymer arrays on the substrates of **a** glass, **b** Si, **c** SiO_2/Si, **d** PI and **e** PDMS. Scale bar: **a**-**e** 20 μm.

Supplementary references

1. Choi, H. H., *et al.* Critical assessment of charge mobility extraction in FETs. *Nat. Mater.* **17**, 2 (2017).

2. Dong, H., *et al.* 25th Anniversary Article: Key Points for High-Mobility Organic Field‐Effect Transistors. *Adv*. *Mater*. **25**, 6158 (2013).

3. Feng, J. G., *et al.* “Capillary-Bridge Lithography” for Patterning Organic Crystals toward Mode-Tunable Microlaser Arrays. *Adv. Mater.* **29**, 1603652 (2017).

4. Zhao, Y., *et al.* Regulated Dewetting for Patterning Organic Single Crystals with Pure Crystallographic Orientation toward High Performance Field-Effect Transistors. *Adv. Funct. Mater.* **28**, 1800470 (2018).

5. Stoumpos, C. C., *et al*. Ruddlesden-Popper Hybrid Lead Iodide Perovskite 2D Homologous Semiconductors. *Chem*. *Mater*. **28**, 2852-2867 (2016).

6. Adireddy, S., Lin, C., Cao, B., Zhou, W. & Caruntu, G. Solution-Based Growth of Monodisperse Cube-Like $BaTiO_3$ Colloidal Nanocrystals. *Chem*. *Mater*. **22**, 1946 (2010).
